# Machine learning-driven model construction for automated classification of cognitive styles

**DOI:** 10.3389/fpsyg.2026.1774233

**Published:** 2026-04-09

**Authors:** Xiangwen Wu, Jing Feng, Jianjun Ma, Yu Li, Jiuning Sun

**Affiliations:** 1 Department of Education, Ningxia Normal University, Guyuan, China; 2 Ningxia Key Laboratory of Artificial Intelligence and Basic Education, Ningxia Normal University, Guyuan, China; 3 Guyuan No.4 Middle School, Guyuan, China

**Keywords:** classification models, cognitive style, eye-tracking techniques, machine learning, verbal, visual

## Abstract

Accurate identification of cognitive styles is important for personalized learning environment optimization and human-computer interaction system design. Traditional self-report measures suffer from subjectivity bias, so this study developed a machine learning classification model based on objective physiological data. Focusing on the distinction between verbal and representational cognitive styles, the study collected eye-movement data from 85 participants in a standardized cognitive task via eye-tracking technology. We extracted multidimensional eye-movement features and systematically evaluated the classification performance of six machine learning algorithms: decision tree (DT), K-nearest neighbor algorithm (KNN), plain Bayes (NB), support vector machine (SVM), logistic regression (LR), and integrated learning model (EL). Experimental results show that all algorithms can effectively utilize eye movement features for cognitive style classification, with SVM performing optimally, after optimizing the parameters using the grid optimization method, achieving 82.1% classification accuracy (F1 = 0.715). The method proposed in this study provides a new way for non-invasive assessment of cognitive styles, which can be applied to real-time adaptive learning systems. The research results provide important insights into the development of personalization of educational technology, adaptive design of learning interfaces, and cognitive-perceptual computing systems, and provide valuable references for the fields of educational psychology and human-computer interaction research.

## 1 Introduction

Cognitive style, as a stable pattern of preferences exhibited by individuals during information acquisition, processing, and organization, has a profound impact on learning outcomes and human-computer interaction experiences (Kozhevnikov, 2007). It not only affects how learners acquire, organize, and interpret information, but also directly relates to learning efficiency and the quality of learning outcomes. With the rapid development of educational technology and intelligent learning environments, accurately identifying and adapting to the differences in learners’ cognitive styles has become a core issue in personalized learning research (Pashler et al., 2008). Traditional cognitive style assessments have relied heavily on self-report scales and questionnaires such as Riding and Cheema (1991) Cognitive Style Analysis Test (CSA) and the Felder and Silverman (1988) Learning Style Model (FSLSM). However, there are obvious limitations to these methods: subject self-perception bias, the influence of situational factors, and problems with the reliability of measurement instruments (Peterson et al., 2009). Therefore, it is crucial to explore new methods for cognitive style identification based on objective physiological and behavioral data.

Eye Tracking, as a non-invasive cognitive research tool, has been widely used in recent years in a variety of fields, including psychology, education, linguistics, and cognitive science (Xu et al., 2024). Research has shown that individuals with different cognitive styles have significant differences in visual attention allocation, information search strategies, and processing patterns. As Koć-Januchta et al. (2017) found, Visualizers tend to focus on graphic information and show more complex visual scanning patterns, while Verbalizers focus more on textual content. Tsianos et al. (2009) showed statistically significant differences between learner types, with representational types focusing on visual content, verbal types on text, and intermediates in between. These findings provide an empirical basis for using eye movement data to distinguish cognitive styles. Meanwhile, machine learning techniques have made significant progress in the field of cognitive and behavioral pattern recognition. There have been research attempts to apply machine learning to learning style recognition, such as Özpolat and Akar (2009) use of the NBTree algorithm to classify learner behavior; Graf et al. (2009) predicted learning styles based on learning management system interaction data; Xu et al. (2024) constructed an online automatic classification model for field-independent-field-dependent cognitive styles using machine learning. However, although work has demonstrated that eye-movement data are sensitive to cognitive styles (e.g., field independence/dependence, verbal-visual dimensions), studies have relied primarily on self-report scales or a small number of physiological metrics, and have focused on constructs such as emotion, personality, or expertise, with little research focusing on automatic identification of “verbal-visual” cognitive styles. Automatic identification of “verbal-visual” cognitive styles is still scarce.

This study aims to bridge this research gap by integrating eye-tracking technology with machine learning algorithms to construct an automated cognitive style classification model based on objective physiological data. The main research questions are as follows:

1. How can the objective physiological data obtained from eye-tracking devices be used to extract the key features that can effectively distinguish between verbal and visual cognitive styles?
2. Based on the eye-tracking features, how do different machine learning algorithms perform in the automated classification of verbal - visual cognitive styles?

## 2 Related research

### 2.1 Cognitive style

The term “cognitive style” was first defined by Allport (1937) as “the habitual patterns that an individual exhibits in perception, memory, and thinking activities.” Subsequently, Witkin et al. (1954) proposed the field-dependent/field-independent (FD/FI) dimension through the “stick-frame experiment” to attribute style differences to physiological differentiation of the nervous system. Messick (1976) further states that cognitive styles are “cross-situationally stable, value-neutral processing preferences,” emphasizing their essential difference from ability variables. After the 1980s, the focus of research shifted to information representation modalities, and Riding and Cheema’s (1991) learners with different cognitive styles showed stable differences in encoding preferences for verbal symbols versus visual representations. Verbalizer-Visualizer (V-V) cognitive style, as a key variable in determining an individual’s stabilizing preference in graphic information processing, directly affects students’ acceptance, depth of understanding, and transfer of AIGC-generated content (Ul Ansar and Ganesh, 2023). The Verbalizer-Visualizer dimension of cognitive style is an important classification that describes an individual’s preferred mental representation during information processing. This dimension was originally derived from Paivio (2013) dual-coding theory, which states that there are two separate but interacting information processing channels in the human cognitive system: the verbal system and the pictorial system. Verbalizers tend to use language, words, and symbol systems to represent and process information, have stronger logical expression and abstract reasoning skills, and perform superiorly in tasks such as text comprehension, verbal reasoning, and memory encoding, while Visualizers rely more on pictorial, spatial, and visual patterns to organize cognitive content, and show a higher sensitivity and processing efficiency in graphic, diagrammatic, and spatial tasks (Koć-Januchta et al., 2017; Tsianos et al., 2009). On this basis, Richardson (1977) developed the Verbal-Visual Quotient (VVQ), which can be used to measure an individual’s tendency to process in this dimension. Riding and Cheema (1991) further included it as one of the two core dimensions of cognitive style, and in a follow-up study, clearly distinguished significant differences between Verbalizers and Visualizers in information processing. Verbalizers usually adopt a linear, sequential, semantic-dominant information processing approach, preferring reading, verbal representation, and logical deduction, while Visualizers are accustomed to integrating information in a holistic, structured, image-dominant manner, and are more adept at spatial thinking, graphic integration, and multimodal learning tasks (Kozhevnikov et al., 2002). Xue and Zeng (2020) and others further empirically demonstrated this difference based on eye movement experiments: in the graphic learning task, Visualizers significantly gazed more at the image area, while Verbalizers’ gaze behavior was focused on the text content.

### 2.2 Relevant applications of eye tracking technology and cognitive style

Eye-tracking technology can provide objective data on key cognitive processes such as attention allocation, information processing, cognitive load, and learning strategies by recording an individual’s eye movement trajectory during a visual task (Xue and Li, 2021) They usually include dimensions such as gaze point, gaze duration, eye hopping path, and pupil diameter, which are effective indicators of an individual’s attention allocation and information processing strategies when facing a cognitive task (Šola et al., 2024) For example, Xue and Zhu (2024) found that field-dependent (FD) learners typically show more dispersed gaze points and more sweeping behaviors, whereas field-independent (FI) learners tend to have a more focused and directed pattern of visual attention; Significant relationships between eye movement characteristics (e.g., total number of gazes, mean gaze duration, etc.) and cognitive performance on the Ruff Figure Fluency Test (RFFT) (Steichen and Fu, 2020); Henderson et al. (2013) found that eye movement pattern categorization accurately predicts a person’s cognitive state in different tasks, including reading, pseudo-reading, scene search, and scene memory; Steichen and Fu (2020) showed that users’ cognitive styles can be effectively predicted by eye-tracking techniques, and their results showed that the accuracy of classification based on eye-movement features was as high as 86%, with features such as the length of the sweep and the graphical AOI (area of interest) contributing the most to the classification. Some scholars have also explored the use of multimodal approaches to identify cognitive styles. For instance, Zhu (2024) selected physiological data from three modalities - eye movement, electroencephalogram (EEG), and facial expression - which are closely related to learning cognition. Through this, he explored the relationship between the online learning outcomes of learners with different cognitive styles and the data from each of these modalities during the online learning process. Xue et al. (2024) constructed an intelligent recognition framework for cognitive style based on multimodal data by extracting EEG data, expression data and eye movement data generated in learners ‘online learning, and used six machine learning models to verify its effectiveness.

### 2.3 Machine learning

Machine Learning (hereinafter referred to as ML) is a class of techniques that allow computers to automatically extract patterns from data and make predictions or decisions through algorithms, the core paradigm of which is supervised learning: given a labeled training set, the model learns a mapping function between input features and labels to classify or regress new samples (Mitchell, 2003). Machine learning methods are a way for computers to use existing data (experience) to come up with some kind of model and use those models to predict the future (Sun and Huang, 2017). In most research scenarios of cognitive style recognition, traditional machine learning is a more reliable and suitable first choice. Only in the specific scenarios of large samples, strong engineering implementation requirements, and high-dimensional original eye tracking data, deep learning has significant advantages (Amiri et al., 2024). And in classification tasks, algorithms such as Decision Tree (DT), K-Nearest Neighbor (kNN), Naïve Bayes (NB), Support Vector Machine (SVM), Logistic Regression (LR), and Ensemble Learning (EL) have been repeatedly validated to capture high-dimensional, nonlinear, and noisy data (Pradnya Sidhawara et al., 2020). Thanks to its powerful feature learning and generalization capabilities, ML has become a mainstream tool in psychological and behavioral sciences: Chui et al. (2020). Predicting at-risk and borderline students based on a simplified training vector-based support vector machine (RTV-SVM). Rizzo et al. (2022) proposed a machine learning method based on eye-tracking data for detecting cognitive interference. Xue and Zhu (2024) constructed an online automatic classification model for field-independent-field-dependent cognitive styles by using a machine learning model to learn the features of six eye-movement metrics, such as gaze duration, scanning distance, and pupil diameter, of which the logistic regression model had an accuracy rate of 89.01. In Hu et al. (2023)‘s study, key contextual factors that can synergistically differentiate between high and low performers, high and medium performers, and low and medium performers in digital reading were identified through the use of Support Vector Machines (SVMs) and SVM Recursive Feature Elimination Methods in order to enhance students’ digital reading literacy (Villegas-Ch et al., 2023). SVM-based emotion recognition model achieves 70% accuracy in classifying emotions in teaching and learning environments by analyzing 22 facial features, and machine learning algorithms to monitor and classify students’ gestures in order to recognize the emotions they convey in teaching and learning environments. These studies have consistently shown that ML can accomplish the identification, classification, or prediction of psychological traits with significantly better performance than traditional statistical methods when dealing with either behavioral data or traditional scale data, demonstrating its validity and generalizability.

## 3 Cognitive model construction based on eye movement data

**Figure 1:**
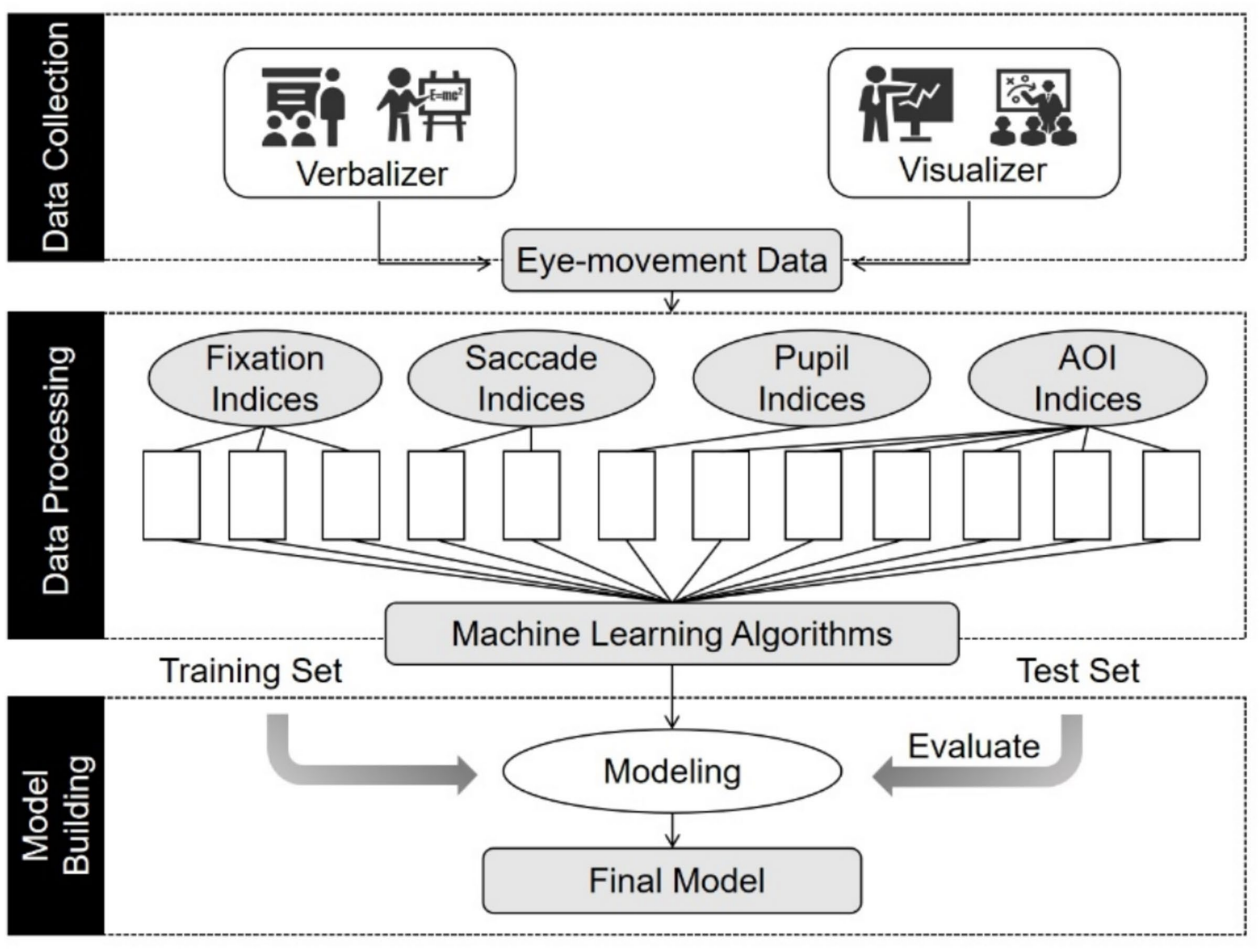
Cognitive style automated classification model.

This study combines eye movement data with machine learning algorithms to construct an automatic recognition model for “verbal-visual” cognitive styles through the paths of data collection, data processing, and model construction, as shown in Figure 1. In the data collection layer, the SR Research EyeLink 1000plus eye-tracking device is used to continuously record the raw eye-movement sequences at a sampling rate of 1,000 Hz while the subjects are reading standardized graphic materials. In the data processing module, spatio-temporal features were extracted from the raw eye movement data collected, and 15 data such as gaze time and number of gaze points, were extracted from four dimensions, such as gaze indicators, which were normalized to obtain 12 variable data such as the percentage of gaze time for statistical convenience. The model construction layer takes the cognitive style label as the target variable, inputs the 12-dimensional features into the machine learning algorithm, and selects the appropriate algorithm to construct the cognitive style automatic classification model. The advantages and disadvantages of the classification effect of the whole cognitive style model mainly depend on the selection of eye movement indexes and machine learning algorithms, on the one hand, the eye movement features provide the model with a highly discriminative source of information; on the other hand, the multi-algorithm comparison ensures that the best generalization performance is obtained under the limited samples.

### 3.1 Selection of eye movement indicators

In this study, we chose the gaze class, sweep class, pupil diameter, and domain-of-interest class features that are commonly used in conducting eye movement studies in education and are closely related to learning cognition (Zhu, 2024). Gaze is a behavior in which the line of sight remains relatively stationary on a specific area of interest (AOI) for a certain period of time, which is an important external manifestation of information processing and cognitive activities. The key indicators of gaze behavior mainly include gaze duration and the number of gaze points. Duration of gaze refers to the length of time that the line of sight remains within a single area of interest (AOI), usually measured in milliseconds (ms). Longer gaze durations typically indicate that individuals are decoding, integrating, or processing information more deeply in the region, reflecting a higher investment of cognitive resources, and tend to be significantly longer when the complexity of the learning material increases or when information needs to be integrated. The number of gaze points is the total number of gaze behaviors in a given area, and when faced with complex or information-intensive learning materials, learners often need to repeatedly visit the same area to extract and comprehend information, resulting in an increased number of gaze points. On the other hand, during prolonged learning or cognitive fatigue, an increase in the number of gaze points may also be observed due to decreased information processing efficiency or reduced ability to maintain attention.

Sweeping is the process of shifting the eyes from one gaze point to another, during which a person’s vision is suppressed, their ability to perceive information is weakened, and they are unable to engage in deep cognitive processing (Yan et al., 2018). Key quantitative indicators of eye-jump behavior include eye-jump duration, number of eye-jumps, and eye-jump distance. Eye-jump duration refers to the time taken to complete a single eye-jump movement itself, i.e., the time interval (usually measured in milliseconds) between the end of one gaze point and the beginning of the next. Longer eye-jump durations are usually associated with longer eye-jump distances. The number of eye jumps refers to the total number of jumping movements of the eye that occur between gaze points within a given task or area. Eye-hop distance refers to the magnitude of a single eye-hop movement, usually measured in degrees of view or screen pixels, and represents the spatial span between the starting gaze point of the eye movement and the target gaze point. More eye hops and longer sweep durations represent less efficient searches (Wu et al., 2019); larger eye-hop distances represent relatively more information gained by the learner in the gaze prior to the eye-hop (Yan and Bai, 2000).

Pupil diameter, the physical size of the pupil, is one of the most important physiological indicators of an individual’s level of cognitive load during cognitive processing. Changes in pupil diameter are influenced by a variety of factors, including ambient light intensity, stimulus color, and emotional arousal. A significant increase in pupil diameter is usually associated with an increase in cognitive load, provided that all these factors are equal. When learners process difficult information or perform complex cognitive tasks, they need to invest more cognitive resources, which is often accompanied by pupil dilation (Cao et al., 2012).

A domain of interest is a regular or irregular area that is artificially delineated when analyzing data and is the area to focus on in a study. By profiling the learner’s gaze behavior within the domain of interest, one can determine the state of attentional focus in that domain of interest. The higher the level of attentional engagement, the greater the allocation of cognitive resources to the domain, and the more focused the learners are on the domain of interest during the learning process (You et al., 2020).

### 3.2 Machine learning algorithm selection

A machine learning algorithm is an algorithmic model implemented by a computer program whose core feature is the ability to automatically learn laws, patterns, or knowledge from data and use these learning results to make predictions, classifications, or decisions about new data without relying on explicit programming instructions. The cognitive load classification model in this study is precisely designed to analyze existing eye movement data to obtain patterns in eye movement behavior of learners with different cognitive styles, so as to make predictions about the cognitive styles of other learners based on the new data. Therefore, in this study, six commonly used machine learning algorithms related to classification are selected based on data type, data size, and other factors, and are operated on eye movement data, respectively, with a view to finding the machine learning algorithms with the best performance. The six machine learning algorithms are Decision Tree (DT), K-Nearest Neighbor algorithm (kNN), Naïve Bayes (NB), Support Vector Machine (SVM), Logistic Regression (LR), and Ensemble Learning (EL).

A decision tree, also known as a Classification Tree, is one of the most widely used and intuitive of the many data mining algorithms. Its working principle is to divide data based on the homogeneity of the data, often using the Entropy or the Gini index as an indicator to build the model.

The K-nearest neighbor algorithm belongs to a type of non-parametric estimation, unlike decision trees, which are algorithms that build models by generalizing the relationships within the data, and do not involve any relationship generalization or estimation of the statistical distribution of the data set (Altman, 1992). The core logic of this algorithm is that similar data fall in close proximity to each other in n-dimensional space and carry the same class labels, so the algorithm will memorize the whole training set as a “dictionary,” and when it encounters new unlabeled data, it will find the existing labeled data that is very close to it in the multidimensional space and never predict the label of the unknown data. When encountering new unlabeled data, the algorithm will find the existing labeled data in the multidimensional space that is very close to it, without ever predicting the label of the unknown data. The main parameter of the K-nearest neighbor algorithm is to set the k-value reasonably. The k-value indicates that the algorithm searches for k known data points with similar distances for an unknown data point, and generally sets the k-value to an odd number (Peterson, 2009). The value of k was set to 5 in this study.

The Plain Bayesian algorithm is a classification method based on Bayes’ law and the assumption of conditional independence of features, with roots in statistics and probability theory. The premise for applying the algorithm is that the variables are independent of each other, and despite the fact that correlations between attributes are inevitable, the model can still perform well (Rish, 2001). It should be noted that when a large amount of data needs to be sampled, random sampling needs to be used to truly reflect the hidden structure within the original data. Whereas for small training sets, the use of Laplace correction is recommended.

A support vector machine is a supervised learning algorithm that is primarily used for classification problems. It operates on a process of constantly fitting a suitable boundary that circles similar data points (i.e., belonging to the same class) and computationally maximizes the spacing between the two classes. Its biggest drawback is that it consumes a lot of computational resources during the modeling process, but once the model is built, it becomes naturally immune to overfitting.

Logistic regression is a statistical learning model for solving binary classification problems and belongs to the category of generalized linear models, which essentially classify samples by constructing probabilistic models. It determines the likelihood that a sample belongs to a certain category by mapping the output of a linear combination to a probability value in the interval [0, 1]. Due to a few parameters and low computational complexity, logistic regression is well-suited for large-scale data and real-time scenarios. However, its handling of nonlinear data has disadvantages, requiring manual construction of higher-order features or the use of kernel methods.

Integrated learning modeling refers to the use of a series of independent models to make predictions and then combining them to create a single model. Individual models are sometimes only locally optimal, or overfitting occurs, while integrating multiple models improves accuracy and produces a better model, which is also known as “meta-learning methods (Dietterich, 2000)”. The integration output session is generally conducted by voting, and the result with the highest number of votes is elected as the final output of the integrated model. Since the accuracy of the various models varies, different weights can also be assigned to each model so that the final prediction accuracy can be improved.

## 4 Experimental design

### 4.1 Material selection and design

In this study, the classical experiments “Principle of Lightning Formation” and “Acquired Helplessness of Dogs” were selected according to the principles of classification of procedural and declarative knowledge, respectively, and the experimental materials were edited with Photoshop 2024 software, with pixels of 1920*1080; the images were displayed with both pictures and text information for learners’ cognitive processing. The experimental materials were edited in Photoshop 2024 with a pixel size of 1920*1080; the images were displayed with both pictures and textual information for learners’ cognitive processing.

### 4.2 Measuring tools

Cognitive style in this study was measured using the Solomon Cognitive Style Inventory (Felder and Spurlin, 1991) and the Verbalizer-Visualizer Questionnaire (VVQ) (Kirby et al., 1988). The Solomon Cognitive Style Scale consists of 11 questions such as “When you recall the events that happened yesterday, which is more likely to come to your mind - an image or some words?” The VVQ scale The VVQ scale consists of 30 questions such as “Do I enjoy working that requires the use of language? Yes or No?” The Solomon Cognitive Style Scale can better reflect the cognitive style of Chinese students, and the VVQ scale was proposed by Kirby in 1988 to measure the cognitive style of verbal and representational learners, which has been proven to have good validity and reliability.

The eye movement data acquisition device used in the study was the SR Research Eye Link 1000plus, which has a sampling rate of 1,000 Hz and an average accuracy of 0.5°. The experimental program was written using the accompanying Experiment Builder (EB) software, while the Data Viewer software was used to analyze the generated data, extracting data such as gaze points and generating the corresponding heat map.

### 4.3 Experimental subjects

Eighty-five college students from a university in Ningxia, aged between 19 and 22 years old, in good health and free from diseases that might affect the experimental measurements, were selected for this study. The subjects’ naked eye or corrected visual acuity was normal, and if they wore eyeglasses, the prescription should not exceed 500 diopters. In order to ensure that the subjects’ attention is sufficiently focused, it is necessary to screen out the group that does not have prior knowledge by means of a pre-test questionnaire; at the same time, in order to ensure that the learning process can be carried out smoothly, the use of the experimental equipment and procedures is explained to the subjects in detail before the start of the experiment. Combining the results of the Solomon Cognitive Style Measurement Questionnaire and the Verbal-Representational Scale, 77 of the 85 subjects had a more pronounced cognitive style, of which 45 were verbal and 32 were visual. All the participants used data from a single eye. This study was conducted in accordance with the ethical standards of the Ethics Review Committee of Faculty of Education, Ningxia Normal University and was approved by the committee. All participants were informed of the purpose and procedure of the experiment before data collection.

### 4.4 Experimental procedures

**Figure 2:**
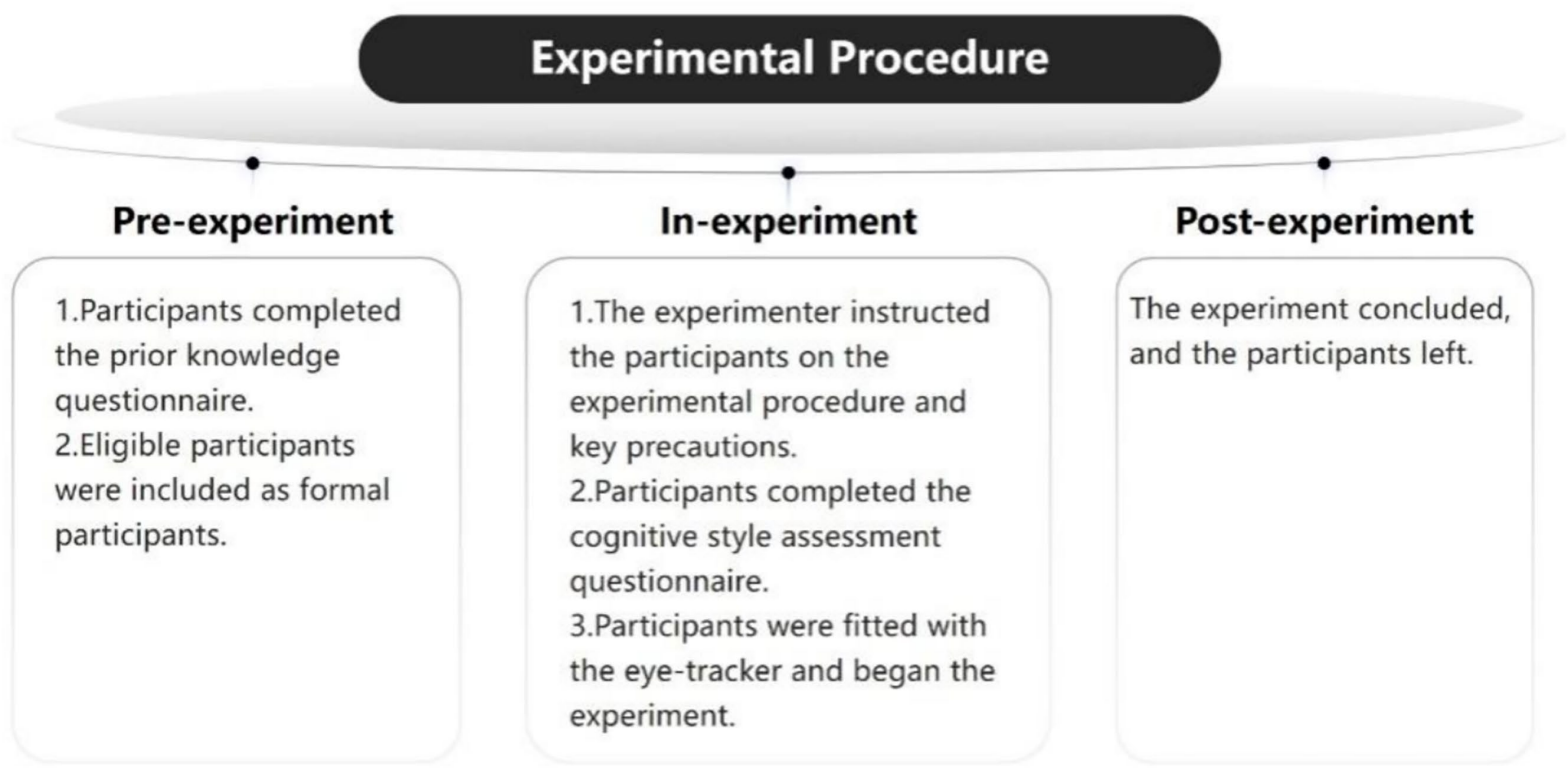
Flow chart of the experiment.

The flow of the experiment is shown in Figure 2. Before the experiment, the subjects filled out the prior knowledge questionnaire, and those who met the conditions were formally included in the scope of the subjects. After entering the laboratory, the master explained the experimental procedure and precautions to the subjects in detail, and the subjects completed two cognitive style questionnaires; the eye-tracking experiment started formally, and the master calibrated the eye-tracking device after the subjects were seated, and those who passed the calibration formally viewed the experimental materials. The experimental materials were two photographs about “the formation principle of lightning” and “the learned helplessness of dogs”; the former was displayed for 1.5 min with a 20-s break, and the latter was displayed for 1 min. During this time, the eye-tracking device continued to record data. At the end of the program, the subjects left and the experiment was completed.

## 5 Data processing

### 5.1 Data preprocessing

After the experiment, a total of 77 sets of valid data were obtained, which contained original data of temporal features such as gaze duration and number of gaze points, and original data of spatial features such as the number of eye jumps and the distance of eye jumps, as well as data related to pupil size and area of interest. In order to facilitate statistics and calculation, the above data were normalized to obtain 12 indicators, such as average gaze duration, as shown in Table 1.

**Table 1:** Normalized eye movement indices.

Normalized eye movement indicator (NEMI)	Calculation method	Unit
Percentage of gaze duration	Length of gaze/total valid viewing hours	—
Mean number of gaze points	Number of gaze points/total valid viewing hours	pcs/s
Mean gaze duration	Length of gaze/number of gaze points	sec/pcs
Mean number of eye beats	Number of eye-rolls/total valid viewing hours	times/s
Mean eye-beat distance	Total eye-beat distance/number of eye-beats	pixels
Mean pupil diameter	Mean of pupil diameters	mm
Picture AOI Percentage of gaze duration	Picture AOI length of gaze/total valid viewing hours	—
Picture AOI Percentage of gaze points	Picture AOI number of gaze points/total number of gaze points	—
Picture AOI Mean gaze duration	Picture AOI length of gaze/number of picture AOI gaze points	sec/pcs
Text AOI Percentage of gaze duration	Text AOI view duration/total active duration	—
Text AOI Percentage of gaze points	Number of text AOI viewpoints/total number of viewpoints	—
Text AOI Mean gaze duration	Text AOI view duration/number of text AOI viewpoints	sec/pcs

### 5.2 Methods of data analysis

After data preprocessing, this study selected six commonly used machine learning algorithms, including an integrated learning model, and used 12 metrics as variables, such as the percentage of gaze duration, and cognitive style type as labels to validate the performance of cognitive style automated classification models. In order to accurately assess the performance of each model, this study adopts the ten-fold cross-validation method, and selects the accuracy rate, precision rate, recall rate, and F1 score as the performance indices of the models.

The ten-fold cross-validation method refers to dividing the dataset into 10 equal parts, using 9 parts for training and one part for testing each time, and repeating 10 times to take the average value. This can effectively avoid sample division bias and is especially suitable for scenarios with a limited sample size. Accuracy rate refers to the ratio of the number of all correctly classified samples to the total number of samples, i.e., the probability of classifying correctly, ranging from 0 to 1. The larger the value, the more accurate the classification carried out using the method. Precision rate is the ratio of the number of samples predicted to be of a certain type that are actually of that type to the total number of samples predicted to be of that type, i.e., the probability of accuracy in predicting a positive class, ranging from 0 to 1, with larger values indicating that the model is more accurate in predicting samples of a positive class. Recall refers to the ratio of the number of samples that are actually of a certain type that are correctly predicted to be of that type to the total number of samples that are actually of that type, i.e., the probability of capturing samples of the positive type, ranging from 0 to 1, with larger values indicating that the model has greater completeness in identifying samples of that type. The F1 Score refers to the reconciled average of the precision rate and the recall rate, which is computed by (the product of the two/the sum of the two)*2, which ranges from 0–1; the larger the value, the better the model’s performance in balancing precision and recall.

## 6 Data analysis

### 6.1 Algorithm validation and presentation of results

**Figure 3:**
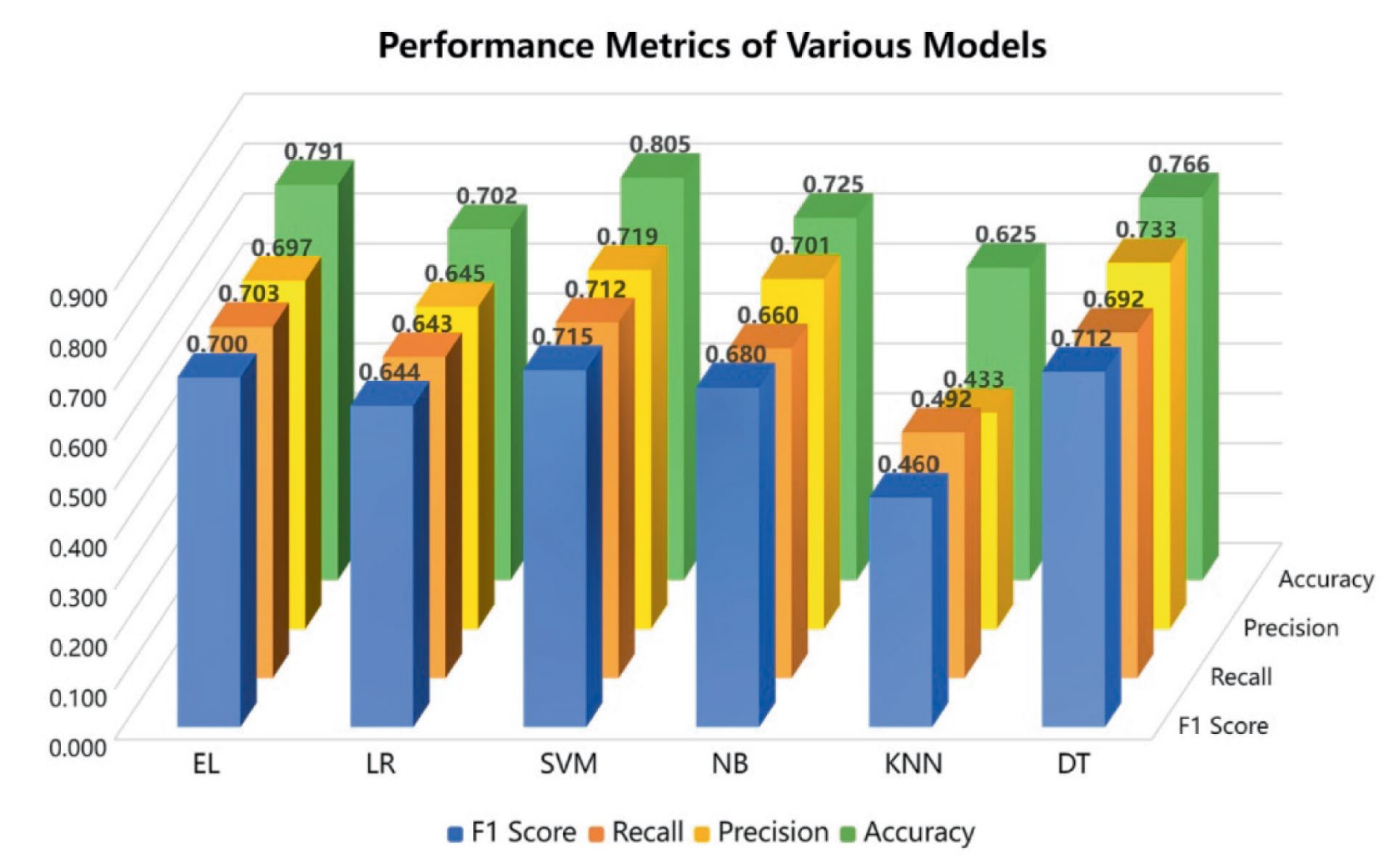
Performance metrics for each model.

The performance of the models constructed by the six selected machine learning algorithms is validated by ten-fold cross-validation, and the performance of each machine learning algorithm model is shown in Figure 3.

Among them, the support vector machine algorithm ranked first with 80.5% accuracy (F1 score of 0.715), and the remaining four algorithms, such as the integrated learning algorithm, had accuracies between 70 and 80%, which were still within the acceptable range. Given the low accuracy of the K nearest neighbor algorithm, it was not included in the construction of the integrated learning model, and only the four algorithms, such as the support vector machine, were used.

Based on the performance results of each algorithm, this study decided to use the Support Vector Machine algorithm and used the Optimize Parameters (Grid) method to adjust the complexity constant C of this algorithm to achieve the best effect. First, set a reasonable range of parameter candidates. Then, through 5-fold cross-validation, traverse all C values as the evaluation criteria for classification performance, and find the C value with the best performance. Finally, use this parameter to train the final model to achieve the best classification effect. Finally, the accuracy of the tuned algorithm model is 82.1%, which is an absolute improvement of 1.6 percentage points. Such an accuracy rate has also been verified in the held-out test set.

### 6.2 Visualization of eye movement indicators for students with different cognitive styles

**Figure 4:**
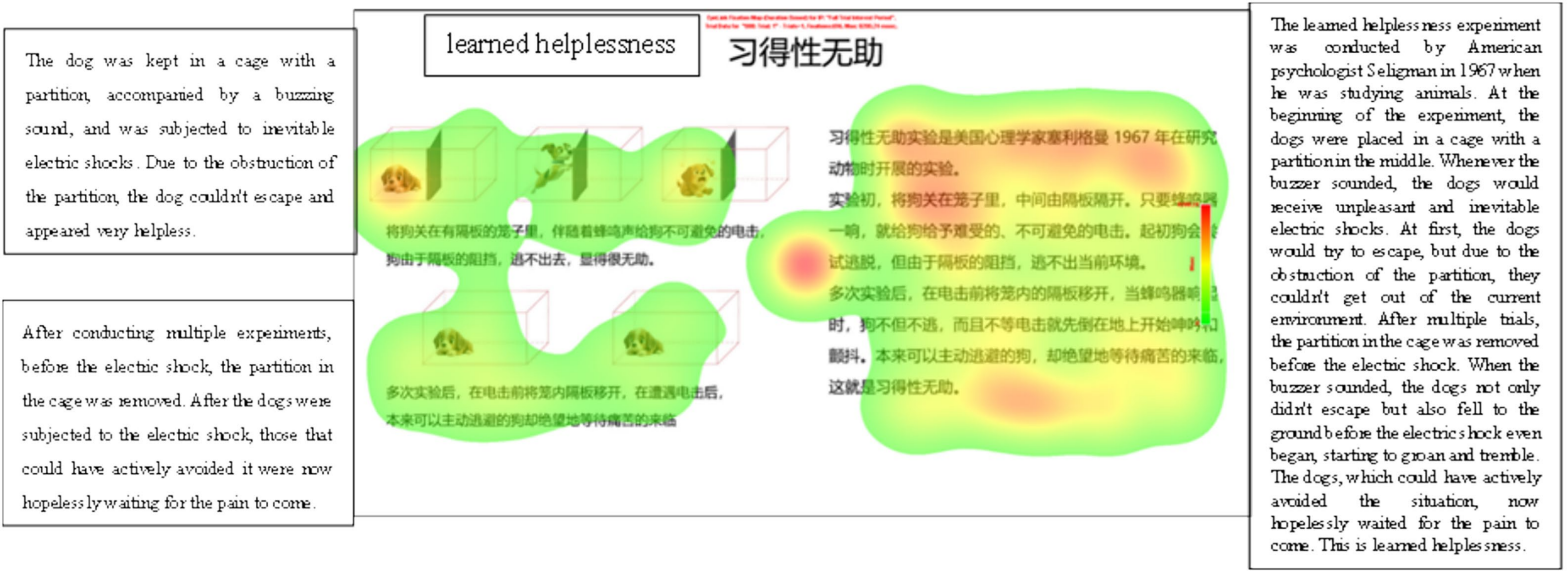
Heat map of eye movements of learners with verbal cognitive style.

**Figure 5:**
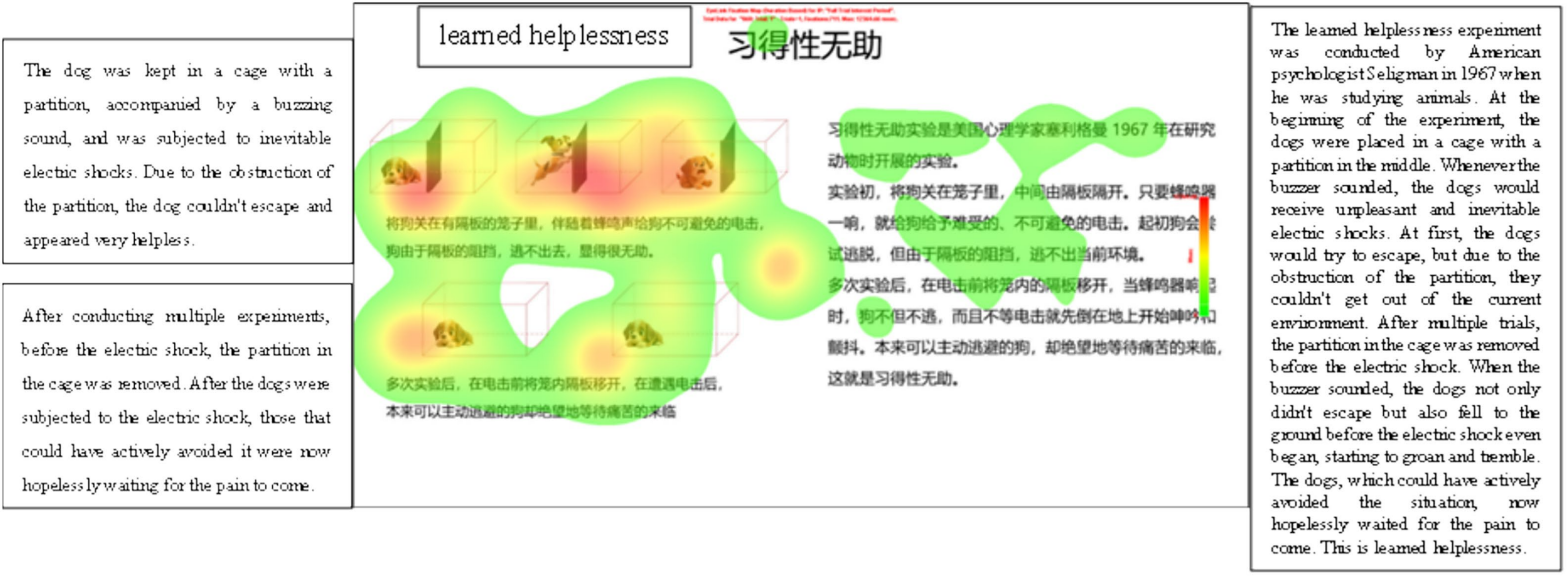
Heat map of eye movements of learners with representational cognitive style.

In order to visualize the differences in eye movement behaviors of different cognitive styles, one subject each of verbal and representational cognitive styles predicted based on the model was randomly selected in this study, and their eye movement data were visualized and analyzed, as shown in Figures 4, 5.

Figures 4, 5 show the eye-movement heat maps of verbal and representational cognitive style learners, respectively, and the darker and brighter areas in the maps represent the areas where the learners’ gaze time is concentrated. It can be seen that verbal learners pay more attention to textual materials, and representational learners pay more attention to pictorial materials. Due to the relatively small sample size of each cognitive style subgroup, separate analyses for participants with different cognitive styles were not conducted in this study.

### 6.3 Correlation of indicators with cognitive style

In order to gain a deeper understanding of the relationship between the indicators and cognitive style, with a view to minimizing the resource cost of cognitive style identification, this study explored the correlation between the eye movement indicators and cognitive style while validating the strengths and weaknesses of the model, as shown in Table 2.

**Table 2:** Correlation of indicators with cognitive style.

Percentage of attention length	Average number of viewpoints	Average gaze duration	Average number of eye jumps	Mean eye-jump distance	Mean pupil diameter
0.202	0.024	0.197	0.025	0.067	0.106
Percentage of attention length within image interest domain	Percentage of viewpoints within the image domain of interest	Average gaze duration within an image’s domain of interest	Percentage of gaze duration within the image domain of interest	Percentage of gaze points within the image’s domain of interest	Mean gaze duration in the image’s domain of interest
0.412^**^	0.418^**^	0.196	0.411^**^	0.423^**^	0.094

**Indicates that *p* is significant at the 0.01 level.

As can be seen from the table, the four indicators of the percentage of attention duration in the picture interest domain, the percentage of attention points in the picture interest domain, the percentage of attention duration in the picture interest domain, and the percentage of attention points in the picture interest domain are significantly correlated with cognitive styles, which is consistent with the previous experience that verbal and representational learners are interested in the textual and pictorial information in the learning materials, respectively. Cognitive style is the external manifestation of learners’ attention preference, thinking style, and information integration logic, so learners with different cognitive styles pay different attention to text and pictures, which leads to a significant correlation between the above indicators and the type of cognitive style. On the other hand, the overall attention percentage and other indicators are affected by attention and some external environmental factors, so there is no significant relationship with cognitive style type. Based on this, the study proposes to focus on the relevant indicators in the interest domain in the construction of the cognitive style classification model, so as to reduce the resource cost in the construction of the cognitive style model.

## 7 Research and discussion

### 7.1 Differences in systematic visual processing patterns between verbal and representational learners

Verbal and visual learners show clearly identifiable and statistically significant differences in systematic visual processing patterns during the performance of standardized cognitive tasks. Through in-depth analysis of eye movement trajectories, heat maps of gaze distribution, and key eye movement indicators, the study reveals fundamental preferences in information acquisition, attention allocation, and cognitive resource investment strategies of individuals with both cognitive styles. For example, verbalizers may be more inclined to process textual information areas in depth, showing longer gaze duration and denser textual area gaze points, while visualizers may show stronger visual attraction and more frequent scanning behaviors for diagrams and image areas. This is consistent with the findings of Koć-Januchta et al. (2017), Tsianos et al. (2009), Xue and Zeng (2020). These differences not only provide an intuitive and interpretable behavioral basis for high-precision classification by machine learning models (especially SVMs), but also strongly empirically support the core idea of cognitive style theory about the existence of intrinsic differences in individuals’ information-processing preferences at the level of dynamic visual behaviors, and provide new experimental evidence for understanding the microbehavioral representation of cognitive styles.

### 7.2 Support vector machines: an effective method for constructing automated classification models of cognitive styles

In this study, multi-dimensional eye movement data such as gaze, eye hopping, pupil reaction, and area of interest (AOI) were systematically collected, from which the most discriminative feature sets were extracted and screened, and 12 core eye movement indicators were formed after normalization. Six classical machine learning algorithms, including Support Vector Machine (SVM), are utilized for model training and comparison. The experimental results show that: the four algorithms, including decision tree and integrated learning model, show good performance, with classification accuracies of more than 70%; support vector machine (SVM) performs the best, and after parameter optimization, the classification accuracy of the model is increased to 82.1%, which is significantly better than the other comparative algorithms in this study, by systematically comparing a variety of machine learning algorithms, we have explicitly confirmed that support vector machine (SVM) is the most important tool for constructing the automated classification models for cognitive styles (verbal vs. visual) in a superior way. There are some limitations in this study, such as single data set, limited sample size, no external validation, robustness test, and class imbalance analysis, which affect the generalization ability of the model and the reliability of the results. The conclusions of this study are only applicable to the current experimental conditions. In contrast, the study by Xue and Zhu (2024) team focused on the field-dependent and field-independent cognitive style dimensions, and they also constructed automated recognition models, but with differences in algorithm selection and feature engineering. Xue’s team evaluated a variety of machine learning algorithms, of which the logistic regression model performed the best, with an accuracy of 89.01%, which was higher than the best performance of SVM in this study. The underlying reason for this difference was:

Firstly, this study focuses on verbal and visual cognitive styles, while Xue’s team studied field-dependent and field-independent cognitive styles. These two cognitive style dimensions are fundamentally different in theoretical conceptualization and behavioral performance. According to Kozhevnikov et al. (2014), field-dependent-field-independent dimensions mainly reflect perceptual organization and cognitive control processes, and their differences in eye-movement patterns are usually manifested in the overall-local attention allocation patterns; whereas verbal-visual dimensions mainly reflect information representation preferences, and their differences in eye-movement features are more often reflected in the gaze preferences for different modal information (text vs. images). This difference in theoretical conceptualization leads to the field-dependent-field-independent dimensions showing more obvious discriminative features on eye-movement data, which enables the machine learning model to achieve higher classification accuracy.

Secondly, Xue’s team found that logistic regression performed best on their dataset, while SVM performed optimally in this study. This difference in algorithmic strengths reflects the distributional properties of different cognitive style dimensions in the feature space. Verbal-representational cognitive styles may have more complex nonlinear boundaries, whereas field-dependent-field-independent dimensions may be closer to linearly divisible feature distributions. As proposed by Riding and Cheema (1991), Richardson (1977), Kozhevnikov et al. (2002), and other scholars, there are significant differences in the knowledge processing tendencies of learners with different cognitive styles.

Despite the differences in specific results between the two studies, both confirm the effectiveness of machine learning methods based on eye movement data in the automated recognition of cognitive styles. Both the 82.1% classification accuracy of this study and the 89.01% of Xue’s team are significantly higher than the randomized classification level, suggesting that eye movement features do contain rich information about cognitive style. The methodological differences and result comparisons between the two studies provide important insights for future research: (1) different cognitive style dimensions may require targeted experimental paradigms and feature engineering strategies; (2) algorithm selection should take into account the feature distribution properties of specific cognitive style dimensions; and (3) multitasking paradigms may provide a more comprehensive cognitive style characterization.

### 7.3 Automated cognitive style classification model enabling intelligent education accuracy

The high-performance cognitive style automated classification model (based on SVM and eye movement features), constructed and validated by the Institute, provides key technical support for advancing the precision and personalization of intelligent education systems. Based on the recognition results of this model, the intelligent education system can dynamically and accurately adapt differentiated learning paths and presentation methods for learners with different cognitive styles. For example, it can provide detailed textual explanations for those with verbal preferences, and rich diagrams, animations, or interactive visualizations and cognitive support strategies for those with representational preferences, so as to truly realize the scale of “tailor-made education.” At the same time, the integration of cognitive style recognition into intelligent guidance systems or teacher decision-making tools can facilitate the formation of a new “human-machine collaboration” education ecosystem, where the technology is responsible for real-time insight and personalized resources, and the teacher can focus on higher-order teaching activities and humanistic care. In the future, the model can be used for the design of multimedia screen languages based on different cognitive styles. Since these models are essentially composed of simple formulas and syntax rules, they can be easily embedded into most programming languages, and there are also many mature tools for deploying the model, which facilitates its application.

## 8 Limitations of the study

Focusing on the automated and objective recognition of cognitive styles using machine learning techniques, this study successfully constructed and validated a cognitive style classification model based on eye-tracking data. However, there are some limitations in the implementation process, such as: the learner sample characteristics and the types of learning materials in this study are relatively homogeneous, which limits the generalizability and generalization ability of the resulting model in a wider range of populations and diverse learning scenarios to a certain extent; and the current model mainly relies on eye-tracking behavioral data as the input features. Although eye movements are an important window to reflect cognitive processes, relying only on single-modal data may not fully capture the full complexity of cognitive styles. In the future, we may consider integrating multimodal physiological and behavioral data with functional near-infrared spectroscopic imaging (fNIRS) and electroencephalography (EEG) to construct a richer and more discriminative feature system, with the aim of portraying cognitive styles more comprehensively and accurately, and to improve the explanatory and predictive accuracy of the model. In future research, we will also use external test sets to test robustness under noisy or varying conditions, and evaluate runtime performance, computational cost, scalability, or operational feasibility to fill in the gaps of actual deployment.

## Data Availability

The raw data supporting the conclusions of this article will be made available by the authors, without undue reservation.
