# Supplementary material for: Machine learning-driven model construction for automated classification of cognitive styles
Source: Front Psychol. 2026 Apr 9;17:1774233. doi: 10.3389/fpsyg.2026.1774233 (PMC13103944; doi:10.3389/fpsyg.2026.1774233)
Supplement: Supplementary file 1 [file Data_Sheet_1.PDF]

Attachment

Speech-representational Cognitive Style Measurement Scale(Verbalizer-Visualizer Questionnaire)

quiz	Yes	No
1.I like jobs that require the use of language.		
2.Sometimes the dreams I have are very vivid, making me feel as if I had really experienced them.		
3.I find that explanations can help me read.		
4.I can easily think of synonyms for words.		
5.I can easily think of synonyms for words.		
6.If I have only been to a certain place a few times, it is very difficult for me to build a mind map of this place.		
7.I don't like word games such as word fill-in-the-blank.		
8.I don't believe anyone can think based on mental images.		
9.I like learning new vocabulary.		
10.I often dream of the places I want to go.		
11.I think the new city map is very useful for finding directions.		
12.My imagination is higher than that of ordinary people.		
13.I read very slowly.		
14.As the saying goes, "A painting is worth a thousand words," which is very true for me.		
15.I never daydream.		
16.I have always hated jigsaw puzzles.		
17.I don't like looking up examples in a dictionary.		
18.I never use tables to explain things.		
19.The dreams I had were extremely vivid.		
20.I'd rather read the instructions to know how to operate than be taught by others.		
21.I like news reports with charts.		
22.I never daydream.		
23.I use words more fluently than the average person.		
24.I don't like books with pictures or charts.		
25.I like daydreaming.		
26.When I read books with pictures, I often refer to the pictures.		
27.I can't remember the lyrics well.		
28.I spend very little time increasing my vocabulary.		

29.It's very hard for me to remember my dreams.		
30.The dreams I had were clear rather than blurry.		

Solomon Learning Style Scale

Each of the following questions has two options, A and B. Please choose the option that is closer to your actual situation:

1.When you recall experience yesterday, you most likely to think of: A, A picture B, A few words

2. Which way do you prefer to get new information:

A, through pictures, tables, graphics, or maps, etc. B, words or language

3.In a book with a large number of pictures and tables, you might:

A. Look carefully at the pictures and tables   B. Focus mainly on the text

4.You prefer:

A. teacher who presents a large number of figures on the blackboard   B. A teacher who spends a lot of time explaining

5.Which information do you remember best

A. What is seen   B. What was heard

6.When you are going to a new place, you prefer to pass through:

A. A map   B. Route instructions described in words

7.During the class, when you see tables or sketches, you often remember more easily:

A. A picture B. What the teacher said

8.When someone tells you certain information, the way you prefer is:

A. Tables or pictures   B. A textual summary describing it

9.When you recall the new friends you met at the party, it's easier for you to remember:

A. Their appearance   B. Their self-introductions

10.Which of the following forms of entertainment do you prefer

A. Watching TV B.reading

11.When you describe a place you have been to, you will feel:

A. It is very easy and can be accurately described   B. There are difficulties and the description is not very clear

PRE-SCHOOL TEST

Please complete the following questions based on your experience and knowledge accumulation. Mark √ if you meet the requirements and × if you don't.

I. The Formation Process of Lightning

1.I often read the weather maps in the newspapers   (   )

2.I can distinguish cumulus clouds from cumulonimbus clouds (   )

3.Do I know what a low-voltage system is   (   )

4.Can I explain how the wind is formed   (   )

5.I know the meaning of this symbol 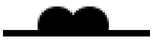 ( )

6.I know the meaning of this symbol 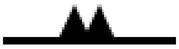 ( )

II. Learned Helplessness

1.I often read the relevant experiments of psychologist Seligman ( )

2.I clearly know Seligman's experiment on dogs ( )

3.I have systematically studied the learned helplessness experiment ( )

4.I've heard of the learned helplessness experiment ( )

5.I can clearly explain learned helplessness ( )

6.I clearly know the experiment between dogs and electric shock ( )

Post-test questions

Please tick the options you think are correct based on your reading and learning memory just now, such as (yes √/ No), and complete the short-answer questions at the end of each part.

I. The Formation Process of Lightning

1.The upper part of the cloud is composed of ice crystals. (Yes/No)

2.Electric charge is generated due to the friction between water droplets and ice crystals. (Yes/No)

3.Negatively charged particles fall to the top of the cloud (yes/No)

4.The charge known as the "step leader" moves downward along a stepped trajectory (yes/No)

5.The negative charges at the bottom of the cloud freely move towards the ground. (Yes/No)

6.The thunder accompanied by lightning is formed by the collision of electric charges. (Yes/No)

7.Could you briefly describe the formation process of lightning?

II. Learned Helplessness

1.The puppy seemed very helpless when it was electrocuted for the first time (yes/no).

2.The puppy had nowhere to run when it was electrocuted for the first time. (Yes/No)

3.The puppy seemed very helpless when it was electrocuted many times. (Yes/No)

4.The puppy had nowhere to run when it was electrocuted many times. (Yes/No)

5.The puppy was still helpless when it was electrocuted after the partition was removed. (Yes/No)

6.After the partition was removed, the puppy still had nowhere to run when it was electrocuted. (Yes/No)

7.After the partition was removed, the puppy still had nowhere to run when it was electrocuted. (Yes/No)
